# *Harposporium incensis* sp. nov. a South American cordycipitoid species exhibiting inter-phylum host-jumping and having potential as a biological control agent for pest management

**DOI:** 10.1080/21501203.2024.2350959

**Published:** 2024-05-27

**Authors:** Ming-Jun Chen, López-Juan Chavez, Jin-Yuan Kang, Jiang-Xin Hu, Jian-Fei Dong, You-Jiu Tan, Zhu-An Chen, Bo Huang, Chun-Ru Li, Chang-Sheng Sun, Nigel Hywel-Jones, Xing-Zhong Liu, Zeng-Zhi Li

**Affiliations:** a Provincial Key Laboratory of Microbial Control, Anhui Agricultural University, Hefei, China; b Peru Biotech Eirl, Lima, Peru; c College of Life Science, Nankai University, Tianjin, China; d BioAsia Life Science Institude, Pinghu, China

**Keywords:** Ophiocordyceps, *Harposporium*, taxonomy, host jumping, biological control agent

## Abstract

Macro- and microscopic morphological studies and multilocus phylogenetic analysis were made on larval specimens of a ghost moth collected from a pigeon pea plantation in Huánuco, Peru. DNA sequences from the cadaver and the fungal isolates obtained represented a monophyletic clade based on the phylogeny. All morphological characters and molecular data showed that the pathogenic fungus infecting the ghost moth larvae was an unknown cordycipitoid species, herein described as, *Harposporium incensis* sp. nov. based on morphological features and multilocus phylogenetic analysis on the cadaver and fungus isolated from the same specimen. The far-related and ecologically different hosts of teleomorph and anamorph of this new species display a peculiar inter-phylum host jumping between the insect *Trichophassus giganteus* of the phylum Arthropoda and the nematode *Caenorhabditis elegans* of the phylum Nematoda and have biological control potential.

## 1. Introduction

*Ophiocordycipitaceae* is a diverse and widespread family in the *Hypocreales*, established by Sung et al. (2007) based on a multilocus phylogeny analysis of taxa previously considered in the *Clavicipitaceae sensu lato*. Quandt et al. (2014) revised the family and proposed to protect six monophyletic genera across both asexual and sexual life stages, including *Drechmeria*, *Harposporium*, *Ophiocordyceps*, *Polycephalomyces*, *Purpureocillium*, and *Tolypocladium*. But Xiao et al. (2023) established a new family, *Polycephalomycetaceae* including *Polycephalomyces*.

*Harposporium* is a common genus of soil fungi, erected by Lohde in 1874 and emended by Zopf (1888). Like *Drechmeria*, the anamorphs of *Harposporium* are pathogens of nematodes, with some also infecting rotifers or tardigrades (Drechsler 1968; Barron 1977). Duddington (1955) discovered a fungus that lives on nematodes which he identified as *Harposporium anguillulae*. This was the first fungus described as nematophagous at the end of the 19th century (Glockling 1993). For a long period, *Harposporium* was considered to be almost exclusively nematophagous in soil, apart from a few species reported as pathogens of rotifers and tardigrades (Hodge et al. 1997). They have been usually isolated from cow dung, farmland, and other places rich in organic matter (Charles et al. 1996). They usually produce infectious conidia, and several species also produce accessory conidia (Hodge et al. 1997), arthroconidia, and chlamydospores (Glockling 1998). Several new species of *Harposporium* were reported in the last century based on microscopic and macroscopic observations of the anamorphs (Barron 1972; Barron and Szijarto 1991) when new species were identified mainly by observation of macroscopic and microscopic sexual (Chaverri et al. 2005) and/or asexual sporogenous structures and spores. Phylogenetics was not used for the systematics of *Harposporium* until early this century (Chaverri et al. 2005). Currently, 38 names are accepted by www.indexfungorum.org, and 37 are accepted by www.speciesfungorum.org. Among them, only 20 species have been cultured on artificial media (Wang et al. 2007). *Harposporium* has been rarely associated with arthropods, notably insects. Samuels (1983) linked it with arthropod hosts when he isolated *H. anguillulae* Lohde from a millipede. Then Shimazu and Glockling (1997) discovered the first case of insect hosts: A longhorn beetle (*Coleoptera*: *Cerambycidae*) infected by a new species, *Harposporium janus*, bearing the synnemata of its *Hirsutella* synanamorph. Evans and Whitehead (2005) found that *Harposporium bredonense* infected the larvae of a longhorn beetle while its synanamorph, *Hirsutella* sp., infected the larvae of a closely related histerid beetle (*Coleoptera*: *Histeridae*); Main synnemata of *H. bredonense* arose from the posterior of the host abdomen, while *Hirsutella* synnemata were produced at the anterior part of the host and *in vitro*. Meanwhile, Chaverri et al. (2005) found a *Cryptorhynchus* weevil (*Coleoptera*: *Curculionidae*) infected by a species of *Podocrella*, which was determined to be the teleomorph of a *Harposporium*, while the hosts of the other three *Podocrella* species were all unknown arthropods. Under the frame of *One Fungus, One Name*, the teleomorph name *Podocrella* was combined with the earlier anamorph name *Harposporium*, together with *Atricordyceps, Polyrhina*, and *Wakefieldiomyces* (Spatafora et al. 2015). These findings of *Podocrella* spp. indicated that the teleomorphs of *Harposporium* species might often be associated with insects or other invertebrates. However, all these associations are those of anamorph or teleomorph to just the same host species, suggesting that *Harposporium* spp. might be host-specific. It has never been observed that a pair of anamorph and teleomorph share hosts of different invertebrates.

Some specimens of infected ghost moth larvae were sent from Peru for identification. The signs of their typical capitate-stipitate stromata with a unique dark discoid head suggested a possible *Podocrella*-like affiliation with *Ophiocordycipitaceae*. However, no typical stromata have ever been observed with the four known *Podocrella* spp. because their stipes were buried in wood (Chaverri et al. 2005). The isolates from the specimens further proved phylogenetically that the anamorph was an unknown *Harposporium* species.

## 2. Materials and methods

### 2.1. Sample collection and isolation

**Figure 1.**
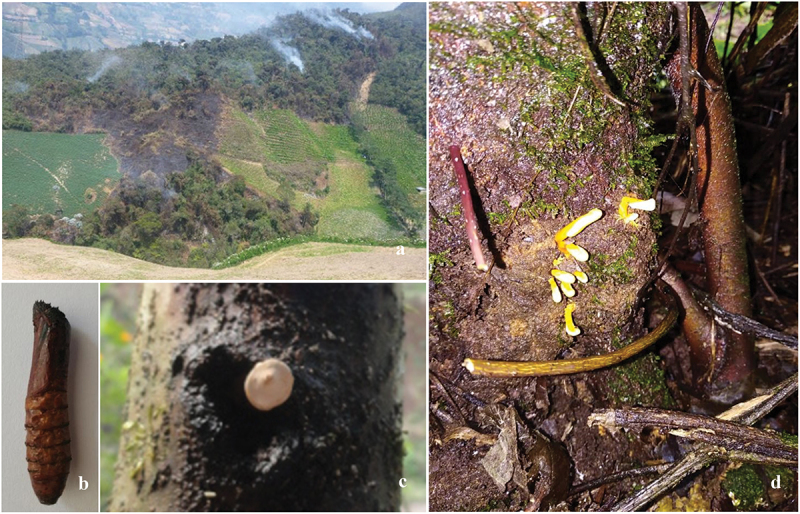
Ecological and field characteristics of the pigeon pea ghost moth, *Trichophassus giganteus*. (a) Aerial view of pigeon pea plantations in Chinchao, Huánuco, Peru. (b) A pupa of the ghost moth. (c) Top of a fruiting body of *Harposporium incensis* emerging from ghost moth pupae in tunnels on the trunk of pigeon pea. (d) Fruiting bodies emerging from ghost moth pupae in tunnels under the cover of frass.

Surveys of entomopathogenic fungi were made in February 2015 at a plantation of pigeon pea, *Cajanus cajan*, a luguminosa dungarunga in Chinchao District, Huánuco Province of Peru (Figure 1). Larval cadaver specimens were collected from the tunnels under wood debris covers on the trunk of pigeon pea, and identified through a mitochondrial cox1 gene sequencing (not described in the present paper) as the cajan ghost moth, *Trichophassus giganteus*. The cadavers were placed separately in plastic boxes, brought to the laboratory for rehydration in a humidity chamber and checked for ascospore discharge every 2 hours under an Olympus SZX16 stereomicroscope. The discharged ascospores around fruiting bodies on glass slides were carefully removed to PDA plates with a flame-sterilised inoculation pin. The cultures were incubated at 25 °C for 2 weeks under 12 h-light/12 h-dark conditions. The specimens were deposited at Zhejiang BioAsia Herbarium (ZBAH) and the isolate is stored at Zhejiang BioAsia Culture Collection Center (ZBAC), Pinghu, China.

### 2.2. Morphological studies

#### 2.2.1. Teleomorph

The macroscopic morphological characteristics of the specimens were photographed using a digital camera. The stromata and perithecia were checked and dissected in water under an Olympus SZX16 stereo microscope; small pieces of material were torn from stromata, mounted in lactophenol cotton blue, and examined for perithecia, asci, and ascospores under an Olympus B×51 microscope. All the structures were measured and photographs were taken. The measurement and calculation method follow Kuephadungphan et al. (2020).

#### 2.2.2. Anamorph

Growth rates of the colonies were determined from colonies developing on petri dish cultures incubated at 25 °C for 14 d. Detailed colony descriptions and morphological comparisons of fungal structures were determined from cultures on PDA, one-quarter strength SDAY/4 (Difco) (Bischoff et al. 2009) and Czapek-Dox Agar (Difco) for 14 d at 25 °C. A slide culture was set on a PDA medium on glass slides for viewing developing conidiogenous structures and conidia. However, no sporulation was observed with the above media. Therefore, a medium with nematodes added was used to induce conidiogenesis. Meanwhile, wide-mouth jar cultures were also prepared. One hundred grams of wheat bran medium in 110 mm glass canning jars were inoculated with 50 days old petri dish culture.

Two drops of *Caenorhabditis elegans* suspension were used to inoculate a Nematode Growth Medium (NGM) plate coated with *Escherichia coli* OP50. The nematodes propagated at 25 °C for 4 days producing a large amount of individuals. The nematodes were washed with sterile distilled water. Approximately 1,000 nematodes were inoculated onto each one-week-old PDA and CMA plate to observe conidiogenesis and assess nematode infection. A Zeiss Axiocam 202 Mono microscope was used for photography under bright field illumination.

### 2.3. DNA extraction, sequencing, and alignment

The total genomic DNA extraction was carried out from both dried specimens and cultures using a modified CTAB method (Lee and Taylor 1990) and then extracted DNA was stored in a refrigerator at −20 °C. PCR amplification was performed using Polymerase Chain Reaction (PCR) primer pairs. The primer pair, ITS4 and ITS5 was used to amplify the internal transcribed spacer (ITS) region. The two primer pairs, NS1/NS4 and LR5/LROR were applied to amplify the small (SSU) (White et al. 1990) and the large subunit ribosomal RNA (LSU) (Rehner and Samuels 1994), respectively. Translation elongation factor 1-α (*EF-1α*) was amplified with the primers EF1-983F/EF1-2218 R (Rehner and Buckley 2005). The largest and second-largest subunits of RNA polymerase II (RPB1, RPB2) were amplified with the primer pairs CRPB1/RPB1-Cr (Castlebury et al. 2004) and fRPB2-7CR/fRPB2–5 (Liu et al. 1999). DNA sequence reactions were carried out at Sangon Company (Shanghai, China). The resulting sequences were checked manually for the ambiguous base and ambiguous regions created by insertions and deletions and then submitted to GenBank.

### 2.4. Phylogenetic analyses

All sequences used in this study were assembled in BioEdit 7.0.9.0 (Hall 1999), and multiple sequence alignment was performed by Clustal X (version 2.0) (Larkin et al. 2007). Manual adjustments of sequences were further carried out using BioEdit, adjusted to maximise homology. Multi-loci sequence data were subsequently concatenated in the PhyloSuite® v1.2.1 (https://github.com/dongzhang0725/PhyloSuite).

To determine the taxonomic position of the new species, a phylogenetic tree was constructed using multiple loci ITS, SSU, LSU, *EF-1α*, *RPB1*, and *RPB2* from the specimens and culture of the new taxon. Forty-six other taxa in the *Ophiocordycipitaceae* (*Hypocreales*) for constructing a phylogenetic tree were downloaded from GenBank. And *Cordyceps kyusyuensis* EFCC 5886 and *C. militaris* OSC 93623 were used as the outgroup (Table 1). Maximum Likelihood (ML) analysis was performed using RAxML 7.2.8 (Stamatakis 2006). The GTRCAT model was used for all partitions, following recommendations in the RAxML manual against the use of invariant sites, and a bootstrap analysis with 1,000 rapid replicates was performed to gain relative support for the branches. For the Bayesian analysis, Bayesian phylogenetic inference was performed using MrBayes 3.3.7 (Ronquist and Huelsenbeck 2003). The GTR+I+G model was selected by MrModeltest 2.2 (Nylander 2004) as the best nucleotide substitution model. Four MCMC chains were executed simultaneously for 2,000,000 generations with tree sampling every 100 generations. Finally, phylogenetic trees were visualised and modified using the Interactive Tree of Life (iTOL) (https://itol.embl.de), an online tool (Letunic and Bork 2016).

**Table 1.** Accession numbers, strain numbers and origins of *Harposporium* and related taxa used in this study.

Taxon	Specimen voucher	GenBank accession number					
		ITS	nrSSU	nrLSU	*EF-1α*	*RPB1*	*RPB2*
*Drechmeria* sp.	TNS F18495	-	KJ878937	KJ878901	-	KJ879017	-
*D. balanoides*	CBS 250.82	AJ292414	AF339588	KR857692	DQ522342	-	DQ522442
*D. campanulata*	IMI 356051	AJ292416	AF339592	AF339543	-	-	-
*D. coniospora*	CBS 596.92	AF106018	AF106012	-	-	-	-
*D. glocklingiae*	CBS 101434	AJ292418	-	-	-	-	-
*D. panacis*	CBS 142798	MF588878	MF588890	MF588897	MF614144	-	-
*D. rhabdospora*	CBS 101432	AF375050	-	-	-	-	-
*D. sinensis*	CBS 567.95	AJ292417	AF339594	AF339545	DQ522343	DQ522389	DQ522443
*D. sphaerospora*	CBS 522.80	AJ292413	AF339590	AF339541	-	-	-
*D. zeospora*	CBS 335.80	AJ292419	AF339589	AF339540	EF469062	EF469091	EF469109
*Tolypocladium inflatum*	CBS 567.84	MH861779	-	MH873477	-	-	-
*T. inflatum*	CBS 127142	MH864435	-	MH875875	-	-	-
*T. capitata*	NBRC 106327	JN943317	JN941737	JN941404	-	JN992471	-
*T. japonica*	OSC 110991	JN049824	DQ522547	DQ518761	DQ522330	DQ522375	DQ522428
*T. ophioglossoides*	NBRC 106331	JN943320	-	JN941408	-	JN992467	-
*T. paradoxa*	NBRC 106958	JN943324	JN941730	JN941411	AB968600	JN992464	AB968561
*Hirsutella kirchneri*	ARSEF 5551	KM652161	KM652070	KM652113	KM651997	-	-
*H. necatrix*	ARSEF 5549	KM652164	KM652073	KM652116	KM651999	KM652039	-
*H. thompsonii*	ARSEF 257	KM652182	KM652091	KM652136	-	KM652054	-
*Ophiocordyceps sinensis*	ARSEF 6282	KM652173	KM652083	KM652126	KM652009	KM652048	-
*O. sinensis*	EFCC 7287	JN049854	EF468971	EF468827	EF468767	EF468874	EF468924
*O. variabilis*	OSC 111003	-	EF468985	EF468839	EF468779	EF468885	EF468933
*O. variabilis*	ARSEF 5365	-	DQ522555	DQ518769	DQ522340	DQ522386	DQ522437
*Purpureocillium lilacinum*	CBS 284.36	AY624189	-	-	EF468792	EF468898	EF468941
*P. lilacinum*	CBS 431.87	AY624188	-	EF468844	EF468791	EF468897	EF468940
*P. atypicola*	CBS 744.73	-	EF468987	EF468841	EF468786	EF468892	-
*P. atypicola*	RCEF 3833	-	KJ878913	KJ878879	KJ878960	KJ878993	-
*P. takamizusanensis*	NHJ 3497	GU980036	-	EU369033	EU369014	EU369053	EU369074
*P. takamizsanuensis*	NHJ 3582	-	EU369097	EU369034	EU369015	-	-
*Cordyceps kyusyuensis*	EFCC 5886	-	EF468960	EF468813	EF468754	EF468863	EF468917
*C. militaris*	OSC 93623	JN049825	AY184977	AY184966	DQ522332	DQ522377	-
*Harposporium anguillulae*	Harp1	-	-	OL405614	-	-	-
*H. anguillulae*	ARSEF 5593	-	-	AY636081	-	-	-
*H. anguillulae*	ARSEF 5407	-	-	AY636080	-	-	-
*janus*	ARSEF 5601	AF169198	-	KY591457	-	-	-
*H. bysmatosporum*	Harp2	OL405615	-	-	-	-	-
*H. bysmatosporum*	BCRC 34226	FJ380935	-	-	-	-	-
*H. cerberi*	CBS 129580	MH865377	-	-	-	-	-
*H. cycloides*	ARSEF 5599	-	-	AY636083	-	-	-
*H. harposporiferum*	CBS 213.86	MH861945	-	MH873635	-	-	-
*H. helicoides*	CBS 944.70	MH860014	-	MH871800	-	-	-
*H. helicoides*	ARSEF 5354	-	AF339577	AF339527	-	-	-
*H. peltatum*	ARSEF 5410	-	-	AY636082	-	-	-
*H. harposporiferum*	ARSEF 5472	-	AF339569	AF339519	PQ118747	-	-
***H. incensis*****(Teleomorph)**	**ZBAH1472**	**OQ170826**	**OQ170825**	-	**OQ183327**	**OQ186689**	**OQ186691**
***H. incensis*****(Anamorph)**	**ZBAC1472**	**OQ161195**	-	-	**OQ186688**	**OQ186690**	**OQ186692**

New sequences were shown in bold.

## 3. Results

### 3.1. Molecular phylogeny

Sequences from the specimen ZBAH1472 and strain ZBAC1472 have been deposited in GenBank with the following accession numbers (Table 1): ITS = OQ170826; SSU = OQ170825; *EF-1α* = OQ183327; *RPB1* = OQ186689; *RPB2* = OQ186691 for the teleomorph and ITS = OQ161195; *EF-1α* = OQ186688; *RPB1* = OQ186690; *RPB2* = OQ186692 for the anamorph.

For sequence analysis, an ML tree was constructed using a weighted analysis of partial sequences from multi-gene regions, and the results were largely consistent with those from Bayesian analysis. The combined dataset of 48 taxa consisted of 4,832 bp (ITS 413 bp, SSU 1,099 bp, LSU 891 bp, *EF-1α* 548 bp, *RPB1* 863 bp, and *RPB2* 1,018 bp), with some strains, only parts of the primers were utilised.

**Figure 2.**
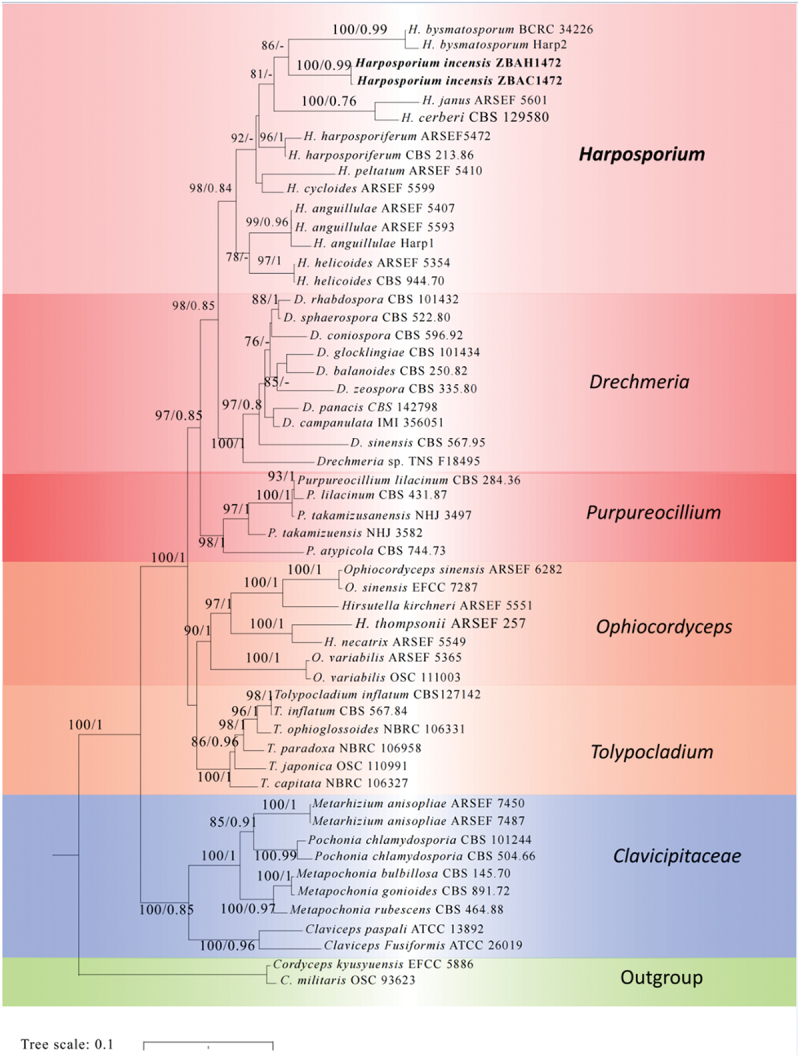
Phylogenetic relationships among *Harposporium* and related genera within *Ophiocordycipitaceae* and *Clavicipitaceae* derived from the combined ITS, SSU, LSU, *EF-1α*, *RPB1*, and *RPB2* sequences based on maximum likelihood and Bayesian analyses. Numbers on the nodes are ML bootstrap values/Bayesian posterior probability above 80% (MPBS-MLBS) or 0.9 (BIPP).

The multi-locus phylogenetic tree (Figure 2) encompassed primarily five genera within *Ophiocordycipitaceae*: *Harposporium*, *Drechmeria*, *Purpureocillium*, *Ophiocordyceps*, and *Tolypocladium*, along with four genera within *Clavicipitaceae*: *Claviceps*, *Metarhizium*, *Pochonia*, and *Metapochonia* in the order *Hypocreales*. *Cordyceps kyusyuensis* EFCC 5886 and *Cordyceps militaris* OSC 93623 of *Cordycipitaceae* were used as the outgroup.

All *Harposporium* species formed a distinct clade with a high bootstrap value and constituted a sister clade to the genus *Drechmeria* which were both apical in the *Ophiocordycipitaceae*. Specifically, *H. incensis*, is introduced as a new species in this study, forming a monophyletic clade comprising its teleomorph ZBAH1472 and the isolate ZBAC1472, exhibiting high support (MLBS = 99.2%, BIPP = 1.00). This cluster further represented a core clade within *Harposporium*, demonstrating strong support (MLBS = 99.2%, BIPP = 1.00). Consequently, the phylogenetic data unequivocally endorse the classification of *H. incensis* as a distinct species within the genus *Harposporium* and as a phylogenetic sister species of *H. bysmatosporum*, *H. janus*, *H. cerberi*, *H. cycloides* as well as *H. harposporiferum*.

## 3.2. Taxonomy

**Figure 3.**
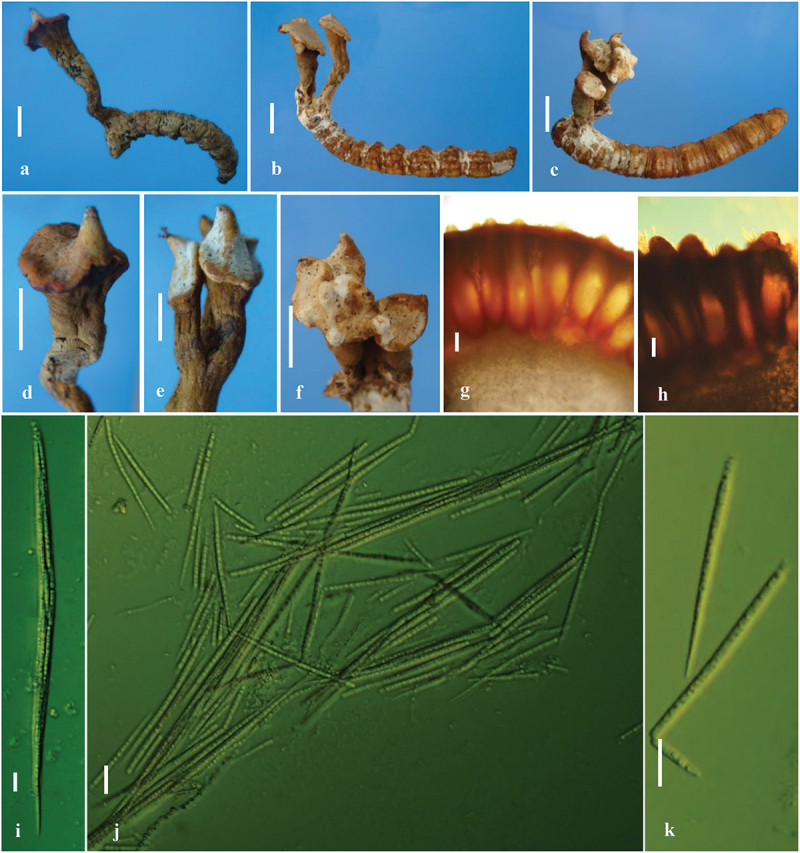
Teleomorph of *Harposporium incensis*. (a–c) Fruiting bodies from larvae of hepialid. (d–f) Perithecia of *H. incensis*. (g–h) Asci of type specimen for *H. incensis*. (i) An ascus. (j–k) Ascospores of *H. incensis*; (k) A disarticulating ascospore and part spore. Scale bars: a–f = 10 mm, g–h = 50 µm, i–k = 10 µm.

***Harposporium incensis*** M.J. Chen, Z.Z. Li et al., Hywel-Jones, sp. nov. Figures 2–3

MycoBank: MB851243.

**Holotype**: Peru, Huánuco Province, Chinchao District in pigeon pea plantation near Montane Forest, August 2014. On larva of *Trichophassus giganteus* (*Lepidoptera*: *Hepialidae*), Juan Chavez López, holotpye, ZBAH1472, ex-holotpye living culture ZBAC1472-DC1.

**Etymology**: After the name of the ancient empire “Inca” which mainly covered Peru and Ecuador (South America), circa 13^#^ Century to 1572.

### Description

**Figure 4.**
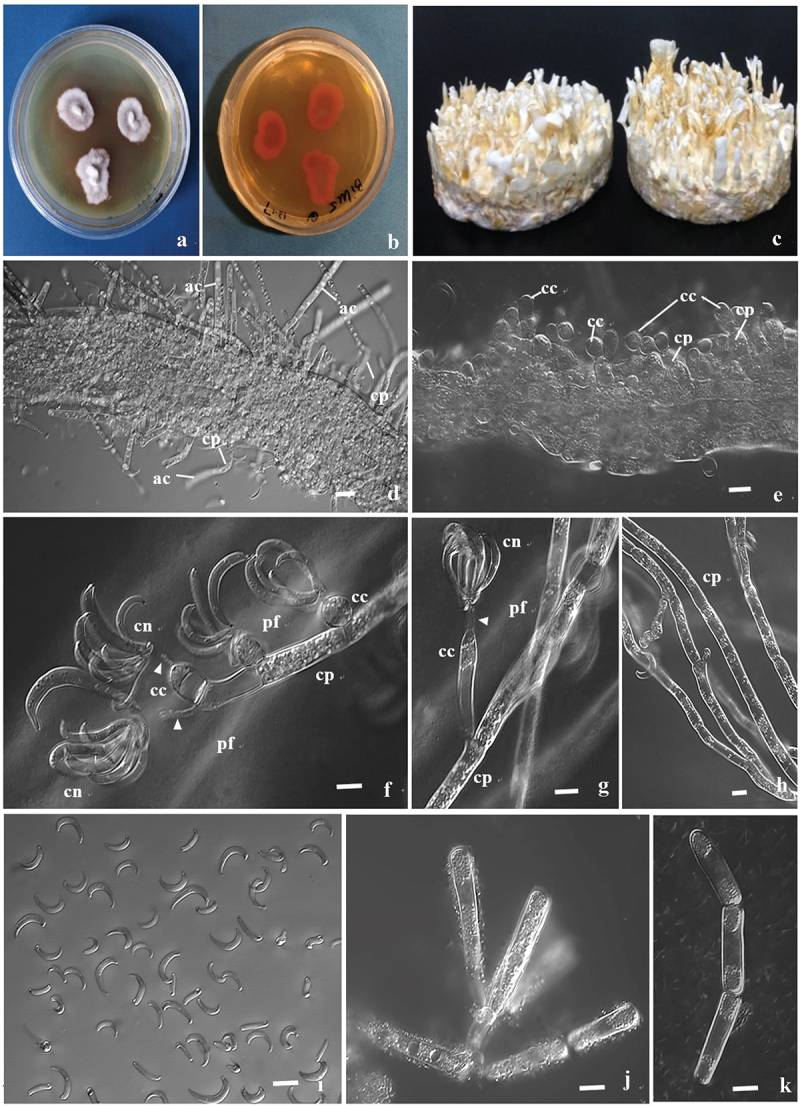
Anamorph of *harposporium incensis*. (a–b) Petri-dish PDA culture on the 21st day; (a) Front side; (b) Back side. (c) Wheat grain culture on the 70th day. (d) Abundant arthroconidia developing on conidia emerging from nematode cuticle. (e) Abundant globose conidiogenous cells developing on conidiophores emerging from nematode cuticle. (f–g) Globose conidiogenous cells (Figure 3f) and a flask-shaped conidiogenous cell (Figure 3g) on conidiophores and crescent-shaped conidia on them. Clusters of conidia are discharged by turgor pressure produced by the high-pressured protoplast. Notice the protoplast streams (indicated by white triangles) ejected from the neck of conidiogenous cells or directly from conidiophores and the protoplast fog of ejected protoplasm. (h) Conidiophores. (i) Conidia. (j–k) Arthroconidia. ac: Arthroconidia; cc: Conidiogenous cell; cn: Conidia; cp: Conidiophore; pf: Protoplasm fog. Scale bars: d, i = 10 µm, e–h, j–k = 5 µm.

**Teleomorph**: On larvae of *Trichophassus giganteus* (*Hepialidae*) (Figure 3a–c), *Stromata* from head or prothorax of the host, 15.3–47 mm long. *Stipes* erect or curved, solitary (Figure 3a–b) or 3–4 branched (Figure 3c) on the upper part, cylindrical, or irregularly distorted, yellowish brown, fleshy, becoming broader upwards, 8–32 × 3–4.5 mm. *Apex* expanding into perithecial disc (Figure 3a–f). *Perithecial disc* terminal, slanted, discoid, brownish, yellowish brown, dark brown to rust brown when dry, 5–14 × 2.5–8 mm, 4 mm thick; sometimes several discs fuse into an irregular fleshy fertile head (Figure 3f), upper surface papillate with ostioles of perithecia (Figure 3g–h). *Perithecia* 547–640 × 132–175 µm, immersed, flask-shaped (Figure 3g–h), closely arranged under upper epidermis, neck slender, forming papillate projections (Figure 3g–h). *Asci* up to 260 × 3–8.4 µm (*n* = 10), 8-spored, hyaline, cylindrical, enlarged middle, tapering to slender tail (Figure 3i), with apical thickened ascus cap, hemispherical, 1.8–3.1 × 2.6–4.2 µm. *Ascospores* multiseptate, filiform, hyaline, smooth, 133.7–219.9 × 2.5–3.6 µm, consisting of cylindrical unicellular segments (Figure 3j), 5.5–9.5 × 2.2–3.7 µm (*n* = 20), disarticulating at the middle septum or the 3/4 septum (Figure 3k). *Part-ascospores* lanceolate, multiseptate, disarticulating once more in the same way into shorter secondary part-ascospores in dry specimens, but, never into the unicellular segments. *Anamorph*: Not seen.

**Culture characteristics**: On PDA slow-growing, ca. 30 mm in after 21 days at 25 °C, white, felt-like, centre raised, peripheral hyphae flocculent (Figure 4a), reverse radiate, reddish-brown (Figure 4b). Reddish-brown pigment was generated around the colony when the white flocculent hyphae grew up to approach the petri dish lid in the later growth. Culture on wheat grain, rope-like hyphae develop into abundant yellow-white sterile syrrotia (Figure 4c) after 70 days at 25 °C, up to 36 mm high, 2.6–3.9 mm thick for single syrrotium, up to 12.8 mm thick for syrrotial bundles.

Conidia absent at 25 °C on PDA or Czapek-Dox media, conidiation on PDA, CMA, and WA after 2–3 days in the presence *of Caenorhabditis elegans* nematodes. *Conidiophores* on PDA septate, 2.4–4.7 µm broad (Figure 4h). *Conidiogenous cells* hyaline, individually on conidiophores or clustered at the apex of conidiophores, mostly ellipsoid (Figure 4d), 5.4–10.2 × 3.5–6.1 µm (average 6.7 × 4.8 µm) (*n* = 50), length/breadth ratio 1.0–1.4, rarely elongating flask-shaped (Figure 4g), 6.7–19.2 × 3.0–3.6 µm (average 10.8 × 4.2 µm), length/breadth ratio 1.6–6.3 (*n* = 5), neck 5.4–9.1 µm long (Figure 4d–f), mainly from conidiophores and some from arthroconidia (Figure 4k). *Conidiophores* (Figure 4h) were cylindric, 3.2–4.2 µm broad. *Conidia* (Figure 4i) discharged by turgor pressure in conidiophores and conidiogenous cells, hyaline, unicellular, falciform, clusters of 4–9 on the neck of conidiogenous cells (Figure 4f), 9.8–19.3 × 0.9–3.2 µm, average 14.4 × 1.9 µm (*n* = 50). *Arthroconidia* (Figure 4j–k) on the apex of conidiophores or apex of branches, cylindric, hyaline, 18.3–64.3 × 2.6–8.5 µm (*n* = 20), usually vacuolate (Figure 4k). Discharged protoplasm around conidia (Figure 4f–g).

**Note**: The phylogeny places *H. incensis* close to *H. bysmatosporum* which was originally described in Colorado, USA (Drechsler 1946). The sequences of *H. bysmatosporum* available from GenBank originated from Morocco (Harp2) and Taiwan, China (BCRC34226). The conidial shape of *H. bysmatosporum* is distinct from that of *H. incensis* – femur-like in the former and falciform in the latter. *H. janus* was the first species to be reliably reported from an arthropod host. Shimazu and Glockling (1997) described *H. janus* from *Anoplophora oshimana* (*Coleoptera*; *Cerambycidae*). The phialide and conidial shape are significantly different from *H. incensis* and no teleomorph has been reported for *H. janus*. There are currently four species of *Harposporium* with known teleomorphs – *H. fuscum*, *H. harposporifera*, *H. peltata*, and *H. poronioides*. *Ophiocordyceps cantharelloides* (Samson and Evans) G.H. Sung, J.M. Sung, Hywel-Jones and Spatafora is possibly also a *Harposporium* based on its overall resemblance to the four teleomorph species and the currently described *H. incensis*. Apart from the lepidopteran host, *H. incensis* differs most significantly from the other species which have ascospores that are less than 100 µm long.

## 4. Discussion

*Trichophassus giganteus*, the host of the teleomorph, is a sizeable hepialid that bores into the trunk of pigeon pea, *Cajanus cajan*, an important crop that is extensively cultivated as a high protein bush legume with a variety of uses in tropical areas of Asia, Africa, and South Africa. It is a food crop of *Leguminosae*, and is also an environmental-friendly plant due to its nitrogen-fixing capacity and enriching soil fertility. In addition, it is also of potential therapeutic value and is used in folk medicine (Morton 1976). Biological control of pigeon pea pests is one of the important strategies of Integrated Pest Management for developing countries (Edpuganti et al. 2018). Potentially, the teleomorph of *H. incensis* as an entomopathogenic morph could be developed into a fungal insecticide for ghost moth control with the anamorph developed into a fungal nematicide. In addition, *Harposporium* spp. are also antagonists to root-knot nematodes and some fungal plant pathogens. In an investigation of antagonistic fungi on tobacco root-knot nematodes in Yunnan, southwest China, 6 isolates of *Harposporium* from the second instars of tobacco root-knot nematodes were identified among 400 isolates from soil and tobacco root samples of a tobacco field (Zhu et al. 2004). Meanwhile, Dai et al. (2022) identified terpendole C, one of seven metabolites from extracts of *Harposporium anguillulae* which exhibited weak nematicidal activity. Furthermore, it is also a potential antagonist against plant diseases caused by *Rhizoctonia*, *Fusarium, Alternaria*, and *Aspergillus*. All of these results figure out the potential of *H. incensis* to be an attractive triple biological control agent (BCA).

The recorded species of *Harposporium* from insects or other invertebrates were all from just one host: an insect or other invertebrate. *H. janus* was recorded from a cerambycid beetle larva (Shimazu and Glockling 1997) and *H. bredonense* from a histerid beetle larva (Evans and Whitehead 2005). Only their anamorphic morphology was recorded, without the teleomorph being observed. By contrast, typical teleomorph morphology was described on hepialid larvae and further detailed dynamic morphology of the anamorph of *H. incensis* on the nematode *C. elegans* were both observed in the present study. All these observations showed a holomorphic life cycle and constituted a clear frame of infection, which will be dealt with in our next paper on this new species.

Noticeably, the hosts of the two morphs of *H. incensis* belong to two distantly related phyla of invertebrates, *Arthropoda* and *Nematoda*. The two morphs are both in the same pigeon pea ecosystem and are both endoparasites, but they have different ecological niches, including microhabitats, trophic modes, ecological adaptation, behaviour, as well as epizootiology. The ghost moth larvae are trunk borers of pigeon pea. They eat phloem and pith of pigeon pea trees, dwell inside tunnels that they bore, and pupate outside the tunnels on the trunk. Meanwhile, the nematode hosts of the anamorphic *H. incensis* dwell in the soil and feed on the roots of pigeon peas. Accordingly, their pathogens also have large differentiations. They are two morphs of the same fungal species and are both endoparasites in the same ecosystem, but they invade their hosts through different entrances: the teleomorph conidia penetrate the integument of host insects by germ tube and appressorium from conidia (Wang and Wang 2017) in tunnels, while the anamorph penetrates the mouth or/and the wall of the gastrointestinal tract by infective falciform conidia (Hodge et al. 1997) in soil. The two morphs dwell in two microhabitats of the pigeon pea ecosystem: trunk and rhizosphere. However, they have a chance to get connected. The disarticulated part-ascospores are forcibly discharged and fall to and scattered in the soil as a resistant stage. When the environment becomes optimal, they germinate and produce falciform conidia through microconidiation, which are swallowed by host nematodes in the soil and cause infection. In this way, the teleomorph meets the hosts of the anamorph in the soil, and host jumping happens there from insects to nematodes, crossing two phyla in the pigeon pea ecosystem via the part-ascospores.

The driving force of the host jumping (Nikoh and Fukatsu 2000) is possibly because narrow host range of *H. incensis* restricted to the pigeon pea ghost moth for its teleomorph or the nematode for its anamorph diversified to cover the nematode and ghost moth, through genetic differentiation of its gene pool as an evolution strategy to escape possible extinction of lineages due to long-term dependency on the ghost moth or the nematode as a particular group of hosts that they colonised (Thines 2019).

In light of epizootiology, *H. incensis* well displayed its persisting form and dispersal forms. Its large and tough sclerotium (cadaver) from the hepialid larvae and ascospores which disarticulate constitutes reliable persisting forms, while enormous numbers of tiny falciform conidia which are ejected by protoplasm discharged due to turgor pressure provide a widely distributed dispersal power. Such mechanisms help the pathogen to persist long in the ecosystem even under adverse climate conditions and survive long in case of host shortage, not to mention extinction. These traits make *H. incensis* a good BCA.

In the present study, *H. incensis* grew in pure culture and formed abundant syrrotia, but without conidiophores and conidia being produced. However, presumably, if nematodes are added to infest the culture, the sterile syrrotia will be fertilised, forming fertile synnemata to produce conidia, which are the infective unit to nematodes and necessary for fungal nematicide development. However, a practical problem for pesticide development (Moosavi et al. 2020) is how to mass infest the culture with nematodes for the mass production of large quantities of biocontrol agents. In addition to the falciform conidia, infective to nematodes, what the conidia infective to pigeon pea ghost moth are to be studied: if they are the falciform conidia infective to nematodes? Additionally, addressing the question of which field inoculum is suitable and developing methods for their mass production is crucial for insect control before initiating a biological control programme.
